# Garlic ameliorates atherosclerosis by regulating ferroptosis pathway: an integrated strategy of network pharmacology, bioinformatic and experimental verification

**DOI:** 10.3389/fphar.2024.1388540

**Published:** 2024-07-23

**Authors:** Tingting Gao, Siqi Gao, Heng Wang, Shule Wang, Lizheng Li, Jie Hu, Sheng Yan, Ruijing Zhang, Yun Zhou, Honglin Dong

**Affiliations:** ^1^ Department of Vascular Surgery, The Second Hospital of Shanxi Medical University, Taiyuan, China; ^2^ Department of Nephrology, The Second Hospital of Shanxi Medical University, Taiyuan, China; ^3^ Shanxi Province Integrated Traditional and Western Medicine Hospital, Taiyuan, China

**Keywords:** garlic, atherosclerosis, network pharmacology, experimental verification, ferroptosis

## Abstract

**Background:**

Atherosclerosis (AS) is a chronic arterial pathology and a leading cause of vascular disease-related mortality. Fatty streaks in the arterial wall develop into atherosclerosis and characteristic plaques. Clinical interventions typically involve lipid-lowering medications and drugs for stabilizing vulnerable plaques, but no direct therapeutic agent specifically targets atherosclerosis. Garlic, also locally known as DASUAN, is recognized as a widely sold herbal dietary supplement esteemed for its cardiovascular benefits. However, the specific mechanisms of garlic’s anti-atherosclerotic effects remain unclear.

**Aims:**

This study aims to elucidate the pharmacological mechanisms through which garlic ameliorates atherosclerosis.

**Methods:**

The study identified the major active components and targets of garlic by screening the TCMSP, TCM-ID, and, ETCM databases. Atherosclerosis-associated targets were obtained from the DisGeNET, GeneCards, and DiGSeE databases, and garlic intervention targets were determined through intersection. Utilizing the intersected genes, Gene Ontology (GO) and Kyoto Encyclopedia of Genes and Genomes (KEGG) pathway enrichment analyses were conducted using R software. A garlic component-disease target network was constructed using Cytoscape. RNA-seq datasets from the GEO database were utilized to identify differentially expressed genes (DEGs) associated with atherosclerosis. The target genes were intersected with DEGs and the FerrDb (ferroptosis database). Molecular docking predicted the binding interactions between active components and the core targets. *In vitro* and *in vivo* experiments validated the identified core targets.

**Results:**

The integration of garlic drug targets with atherosclerotic disease targets identified 230 target genes. Intersection with RNA-seq DEGs revealed 15 upregulated genes, including 8 target genes related to ferroptosis. Molecular docking indicated favorable affinities between garlic active components [Sobrol A, (+)-L-Alliin, Benzaldoxime, Allicin] and target genes (DPP4, ALOX5, GPX4). Experimental validation showed that GARLIC reduces the expression of ferroptosis-related genes in AS, suggesting its therapeutic potential through the regulation of ferroptosis.

**Conclusion:**

Garlic ameliorates atherosclerosis by targeting intra-plaque ferroptosis and reducing lipid peroxidation. These findings provide novel insights into the pharmacological mechanisms underlying the efficacy of garlic in treating AS.

## 1 Introduction

Atherosclerosis is a chronic arterial disease, primarily manifesting as ischemic heart disease, ischemic stroke, and peripheral arterial disease, standing as the leading cause of vascular disease-related mortality (Tsao et al., 2023). Global epidemiological studies conducted in 2020 among individuals aged 30–79 indicate that 27.6% exhibit abnormal intimal thickening in the carotid arteries, exceeding one billion individuals. Additionally, 21.1% are diagnosed with carotid plaques, and carotid artery stenosis affects 1.5% of the population (Song et al., 2020). Atherosclerosis is the primary pathological process underlying most cardiovascular diseases. Its pathological foundation lies in a disorder of lipid metabolism, with fatty streaks in the arterial wall gradually advancing to form atherosclerotic plaques. The acute rupture of these plaques triggers local thrombosis, resulting in local narrowing or detachment of the plaque, leading to partial or complete occlusion of the affected artery. Furthermore, potential pathogenic mechanisms underlying atherosclerosis involve imbalances in lipid metabolism and abnormal immune responses, leading to chronic inflammation of the arterial wall (Libby et al., 2019; Vergallo and Crea, 2020). Studies have revealed that ferroptosis, a novel form of regulated cell death, arises from disruptions in iron metabolism, causing an excessive generation of lipid peroxidation and ultimately culminating in cell death. Ferroptosis is implicated in several cardiovascular diseases, such as atherosclerosis, stroke, ischemia-reperfusion injury, and heart failure (Li et al., 2021; Guo et al., 2022).

Identifying effective therapeutic drugs based on the pathogenic mechanisms of atherosclerosis is crucial for preventing and treating vascular lesions. Currently, drugs that lower lipid levels and stabilize plaques are well-documented (Almeida and Budoff, 2019; Li L. et al., 2022); however, their use is associated with increased risks of hepatic and renal damage, as well as other adverse reactions. Therefore, the development of safe and effective new drugs specifically targeting atherosclerosis is of great importance.

With advancements in healthcare and traditional Chinese medicine, an increasing number of these medicines are employed for early atherosclerosis prevention and treatment (Zhang et al., 2021; Liu et al., 2022; Yadav et al., 2024). Garlic, a prominent herbal dietary supplement, is well-known for its extensive positive impacts, particularly in the treatment and prevention of cardiovascular diseases (Quesada et al., 2020; Li M. et al., 2022). Derived from the *Allium Sativum* plant in the Liliaceae family (Wang et al., 2023), garlic possesses active components, including enzymes (e.g.,.alliinase), sulfur-containing compounds (e.g.,.alliin), and compounds generated by alliinase (e.g.,.allicin) (Majewski, 2014). The antioxidant content of garlic inhibits lipid peroxidation, potentially reducing the occurrence of ferroptosis, oxidative stress, and inflammation in the arterial wall, and thus play a protective role against atherosclerosis (Bai et al., 2020; Li M. et al., 2022). Fang et al. provided a comprehensive review elucidating the key roles of lipid peroxidation and ferroptosis in cardiovascular disease (Fang et al., 2023). Current studies are investigating the potential of some Chinese herbal extracts to treat atherosclerosis by inhibiting ferroptosis (Xu et al., 2024). In addition to these findings, recent clinical trials have provided promising evidence of garlic’s cardiovascular benefits (Emamat et al., 2020).

While garlic demonstrates a distinct therapeutic effect in inhibiting atherosclerosis and inflammation, its specific targets and mechanisms in atherosclerosis treatment remain incompletely understood. Grounded in bioinformatic and computer technique, network pharmacology integrates extensive biological information and data to explore multi-target drug mechanisms from molecules to cells to the human body (Nogales et al., 2022). The advantage of network pharmacology lies in analyzing the interaction network of “drug components—targets—diseases,” revealing synergistic effects among multiple molecular drugs. By employing network pharmacology and molecular docking, the active components of garlic in preventing and treating AS were analyzed to develop novel and clinically significant targeted drugs (Stanzione et al., 2021).

**FIGURE 1:**
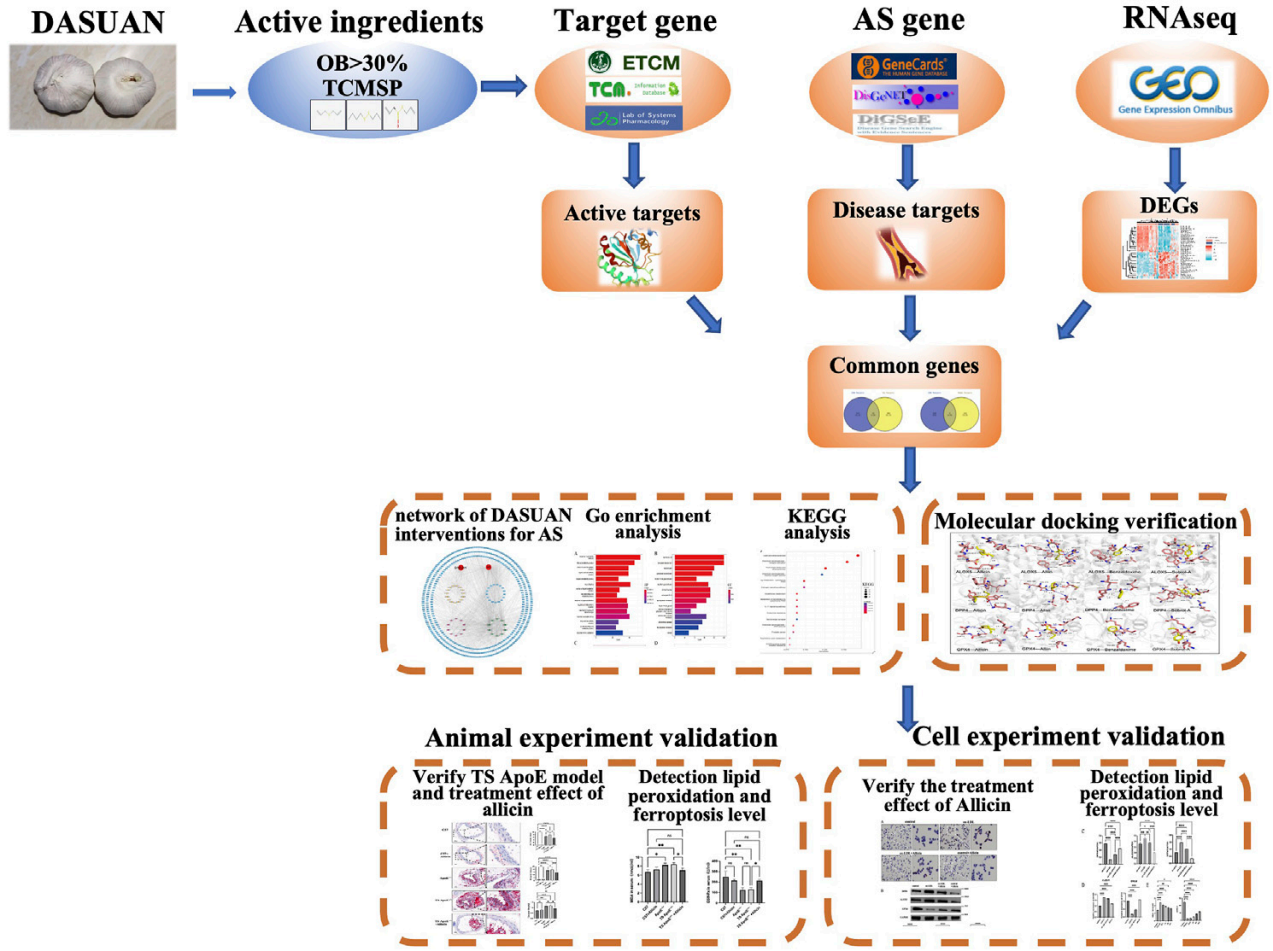
A flow chart illustrated the idea of the project.

This study, based on network pharmacology, molecular docking, bioinformatics, and experimental validation, explores the specific mechanisms through which garlic improves AS. The detailed process of this study is illustrated in Figure 1.

## 2 Materials and methods

### 2.1 Main components and drug targets of garlic

The primary active components of garlic were sourced from the Traditional Chinese Medicine Systems Pharmacology Database and Analysis Platform (TCMSP, https://old.tcmsp-e.com/index.php). Simultaneously, we obtained the pharmacological targets of garlic from TCMSP, Traditional Chinese Medicine Information Database (TCM-ID, https://www.bidd.group/TCMID/), and The Encyclopedia of Traditional Chinese Medicine (ETCM) (Xu et al., 2019). The union of these three databases was considered as the collective set of drug targets associated with garlic.

### 2.2 Atherosclerosis disease targets

Potential targets associated with atherosclerosis were obtained from DisGeNET (Piñero et al., 2020), GeneCards (Safran et al., 2021), and DiGSeE databases (Kim et al., 2013). The search criteria across all databases were based on the keyword “Atherosclerosis.” In DisGeNET, targets were filtered with a criterion of Score gda >0.01; in GeneCards, the criterion was Relevance score >1; and in DiGSeE, the criterion was EVIDENCE SENTENCE SCORE >0.1. The union of targets obtained from these three databases was considered as the collective set of target genes for AS.

### 2.3 Potential targets of garlic in treating atherosclerosis

The potential targets of garlic in treating AS were determined by utilizing the Venny 2.1.0 online plotting (https://bioinfogp.cnb.csic.es/tools/venny/). An intersection analysis was conducted between the targets influenced by garlic and the disease targets associated with AS. This analysis resulted in the identification of 230 target genes, representing the potential targets of garlic in the treatment of AS.

### 2.4 GO and KEGG pathway enrichment analysis

GO and KEGG pathway enrichment analyses were performed using the ClusterProfiler package in the R studio. A total of 230 potential target genes underwent computation. The top 15 GO terms for molecular function (MF), cellular component (CC), and biological process (BP), as well as the top 15 KEGG pathways, were selected for subsequent analysis.

### 2.5 Construction of the network for component-disease-target interactions

The principal active components, potential therapeutic targets obtained through intersection analysis, and the major signaling pathways enriched from GO data were imported into Cytoscape 3.9.1 (Shannon et al., 2003). This facilitated the creation of an interaction network depicting the relationships among components, diseases, and targets. The edges between nodes symbolize the existing relationships.

### 2.6 Expression of key genes in clinical samples of AS

The GSE100927 dataset was sourced from the GEO database, comprising 31 arterial samples from healthy individuals and 65 arterial samples from patients with atherosclerosis (Steenman et al., 2018). This dataset reflects the expression differences in arteries between normal individuals and those with atherosclerosis.

The GSE163154 dataset, also from the GEO database, consists of 16 segments with non-intraplaque hemorrhage (non-IPH) and 27 segments with intraplaque hemorrhage (IPH) in atherosclerotic lesions (Jin et al., 2021). IPH is a critical characteristic of unstable plaques and an independent risk factor for cardiovascular events. This dataset allows for the exploration of differential genes related to plaque stability.

FerrDb is a database related to ferroptosis, monitoring regulators and markers of ferroptosis and ferroptosis-related diseases (Zhou and Bao, 2020). We obtained target genes related to ferroptosis to investigate the mechanistic role of garlic in regulating iron death in AS. Utilizing Rstudio for analysis, we explored the expression patterns of disease-related genes across the mentioned datasets and databases.

### 2.7 Molecular docking and heatmap analysis

The primary active components of garlic were employed as ligands, and crucial genes of interest were selected as receptors for individual molecular docking procedures. The 3D structures of active components were obtained from the TCMSP database, while receptor protein structures were acquired from UniProt and PDB databases. Using PyMol software, the protein files were processed by removing water and ligands to pre-treat the protein structure. Subsequently, these structures were imported into AutoDockTools 1.5.7 (Morris et al., 2008) and transformed into PDBQT format. Molecular docking was performed using AutoDock Vina (Trott and Olson, 2010), and the results were visualized and analyzed using PyMol (Seeliger and de Groot, 2010).

The affinity of molecular docking was represented by molecular binding energy, where values below 0 kcal/mol were considered to indicate some degree of binding capability. Smaller numerical values indicated stronger binding strength. The main active components and target genes were imported into MeV 4.9.0 for heatmap analysis.

### 2.8 Experimental verification

#### 2.8.1 Cell experiments

Murine monocyte-macrophage leukemia cells (RAW264.7) are obtained from Procell Life Science&Technology and cultured in DMEM high glucose medium containing 10% FBS antibiotics (100 U/mL penicillin and 0.1 mg/mL streptomycin) and maintained in a 37°C, 5% CO_2_ incubator. The optimal concentration of ox-LDL (yiyuan biotech, YB-002) and allicin (MCE, HY-N0315) for RAW264.7 was confirmed by CCK8 (MedChemExpress, HY-K030) cytotoxicity assay and subsequent experiments were performed. The cell supernatant was subjected to biochemical assays, including MDA and GSH-Px. Oil red O staining (Solarbio, G1262) was performed on Raw264.7. Western blot was performed for qualitative and semi-quantitative analysis of proteins. The antibodies GPX4 (ab125066), ALOX5 (ab169755), DPP4 (ab187048), and GAPDH (ab8245) were purchased from Abcam.

#### 2.8.2 Real-time quantitative polymerase chain reaction (RT-qPCR) analysis

The primers required for this experiment were synthesized by Shanghai Sangon Bioengineering Technology Service Co., LTD. Primers for GPX4, ALOX5 and GAPDH are as follows: GPX4: forward: 5′-ATA​AGA​ACG​GCT​GCG​TGG​TGA​AG-3′, reverse: 5′-TAG​AGA​TAG​CAC​GGC​AGG​TCC​TTC-3′; ALOX5: forward: 5′- CGG​CGA​TGT​CGA​GGT​TGT​CC-3′, reverse: 5′- CGT​CGG​TGT​TGC​TTG​AGA​ATG​TG-3′; GAPDH: forward: 5′-GAC​ATG​CCG​CCT​GGA​GAA​AC-3′, reverse: 5′- AGC​CCA​GGA​TGC​CCT​TTA​GT-3′.

First, the total mRNA of cells was extracted by using the tissue RNA Rapid extraction kit. The purity and concentration of RNA were measured by ultra microspectrophotometer. The mRNA was then reverse transcribed to cDNA using Takara reverse transcription kit. Then, the expression of the target gene was detected by using the real-time fluorescence kit, the detailed steps are shown in the instruction of article number MF797. Finally, the real-time fluorescence quantitative PCR instrument was used for machine detection. The Ct value of GAPDH was normalized, and the relative gene expression level was calculated using the 2^−ΔΔCT^ method.

#### 2.8.3 Animal grouping and establishment of carotid plaque model

Animals: ApoE^−/−^ male mice aged 8 weeks and weighing 26 ± 2 g were purchased from the Nanjing Junke biological. C57BL/6 male mice aged 8 weeks and weighing 23 ± 2 g were purchased from the Nanjing Junke biological. The mice were housed in specific pathogen-free animal rooms of Shanxi Key Laboratory of Cardiovascular Disease Diagnosis, Treatment and Clinical Pharmacology, Shanxi Medical University, with 3 mice in each cage, and the ear tag number was registered. The mice were fed with high fet diet, unlimited water and food, and the bedding was changed every 5 days. All animal experiments strictly follow the management regulations of animal experiments of the Second Hospital of Shanxi Medical University, and are approved by the ethical review Committee of animal Experiments of the Second Hospital of Shanxi Medical University (Ethics record No. DW2023049).

Model establishment and administration: After 6 weeks of high fat feeding, ApoE^−/−^and C57BL/6 mice were anaesthetized by a mixture of nembutal (60 mg/kg) through intraperitoneal injection. The anterior cervical triangles were accessed by a sagittal anterior neck incision and the right common carotid artery was dissected from circumferential connective tissues. A 150 μm copper wire was placed parallel to one side of the common carotid artery. It was ligated to the internal carotid artery with 6–0 Prolene wire at 1 mm and 4 mm from the common carotid artery bifurcation. The copper wire was then withdrawn, resulting in tandem arterial narrowings. Four weeks post-TS (tandem stenosis), animals were euthanized and arterial segments were collected for analysis. The following exclusion criteria were pre-established: mice that did not survive the TS surgery. No mice were excluded from analyses.

The mice were randomly divided into 4 groups (n = 8 in each group): sham operation group, model group, allicin treatment group and negative control group. Allicin was administered by oral gavage once a day at doses of 10 mg/kg from the first day after TS for 4 weeks. After 4 weeks, all animals were anesthetized. The carotid artery and aorta were taken for observation.

#### 2.8.4 Oil red o staining

Mice carotid artery tissues were washed with cold PBS, frozen sections were fixed with 4% paraformaldehyde, stained with oil red o, and the histological changes were observed under an upright microscope.

#### 2.8.5 Biochemical assays

Blood was obtained using the eyeball extraction method and placed in a yellow procoagulant tube. After centrifugation at 3000 RPM, the upper layer of serum was obtained and stored in a refrigerator at −80°C if necessary for long-term storage. Serum concentrations of TC, TG and LDL-c were measured by automatic biochemical analyzer (BS-240VET, Mindray Global). The MDA (A003-1) and GSH-Px (A005) assay kits were purchased from Nanjing Jiancheng Bioengineering Institute. According to the manufacturer’s instructions, serum and cell culture supernatant were used for the detection of MDA and GSH-Px expression.

## 3 Results

### 3.1 The active components and intervention targets of garlic

**FIGURE 2:**
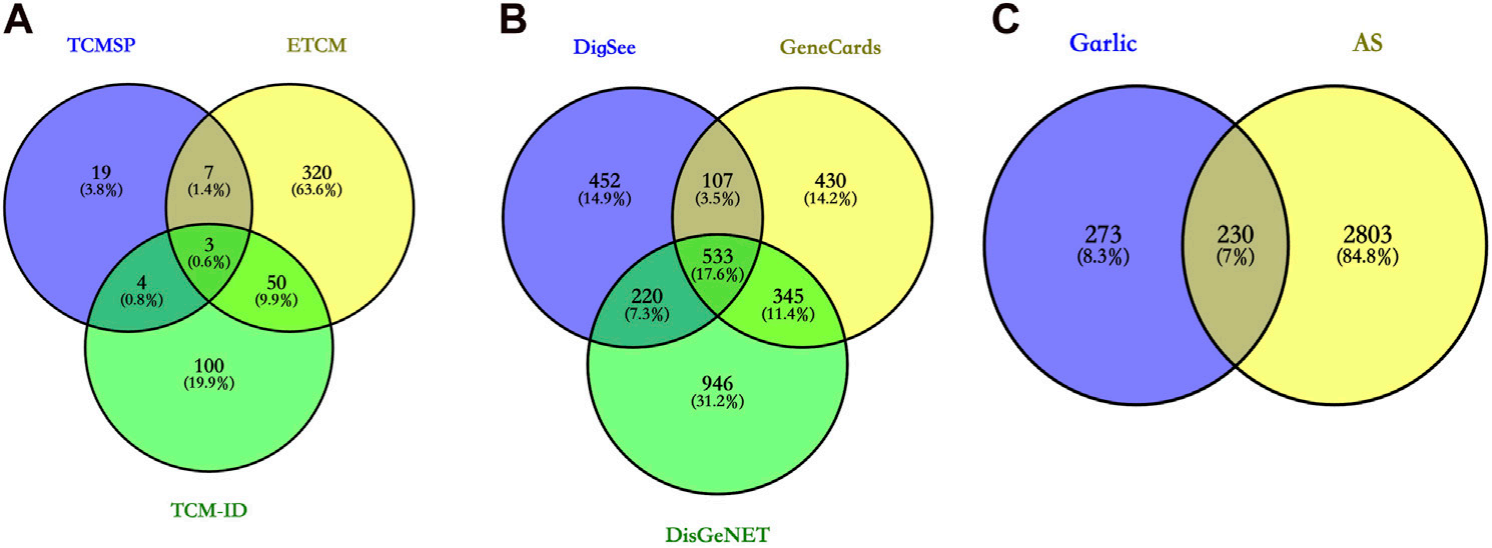
**(A)** The intervention targets of garlic were obtained from three databases, and the total targets were obtained by taking the concatenation. **(B)** Potential therapeutic targets for AS were obtained from three databases, and the total targets were obtained by taking the concurrent set. **(C)** The total intervention targets of garlic were intersected with the total potential therapeutic targets of AS. The potential targets of garlic for the treatment of AS were obtained.

Through literature search, a total of 16 effective components were obtained when the obesity rate (OB) was greater than 30%, and non-specific active components for treatment were excluded (Table 1). Additionally, by collecting intervention targets of garlic from multiple databases, a total of 503 potential targets were identified (Figure 2A).

**TABLE 1:** The main active ingredients of garlic.

Mol ID	Molecule name	MW	OB(%)
MOL001873	Sobrol A	166.19	64.98
MOL000666	Hexanal	100.18	55.71
MOL007601	(+)-L-Alliin	177.25	86.68
MOL007619	Methyl allyl sulfide	88.19	70.09
MOL007643	Methylallyl disulphide	120.26	73.64
MOL008354	Allicin	162.3	78.41
MOL008358	Oil garlic	114.23	74.81
MOL008365	Propyl n-butyl disulfide	164.37	62.95
MOL008370	2,5-Dimethylthiophene	112.21	49.64
MOL008372	2-ethyl-1,3-dithiane	148.32	44.35
MOL007621	DMDS	94.22	39.27
MOL007627	diAllS2	146.3	49.28
MOL008356	Benzaldoxime	121.15	32.46
MOL000775	EEE	88.12	45.02
MOL008350	3-Ethenyl-1,2-dithia-cyclohex-5-ene	144.28	44.6
MOL008361	3-METHYL-2-THIABUTANE	90.21	31.49

### 3.2 Potential therapeutic targets for atherosclerosis

From DisGeNET, GeneCards, and DiGSeE databases, a total of 3,033 main targets for AS were obtained (Figure 2B). Subsequently, the intersection of garlic targets with AS-related targets resulted in 230 potential therapeutic targets (Figure 2C).

### 3.3 GO and KEGG pathway enrichment analysis

**FIGURE 3:**
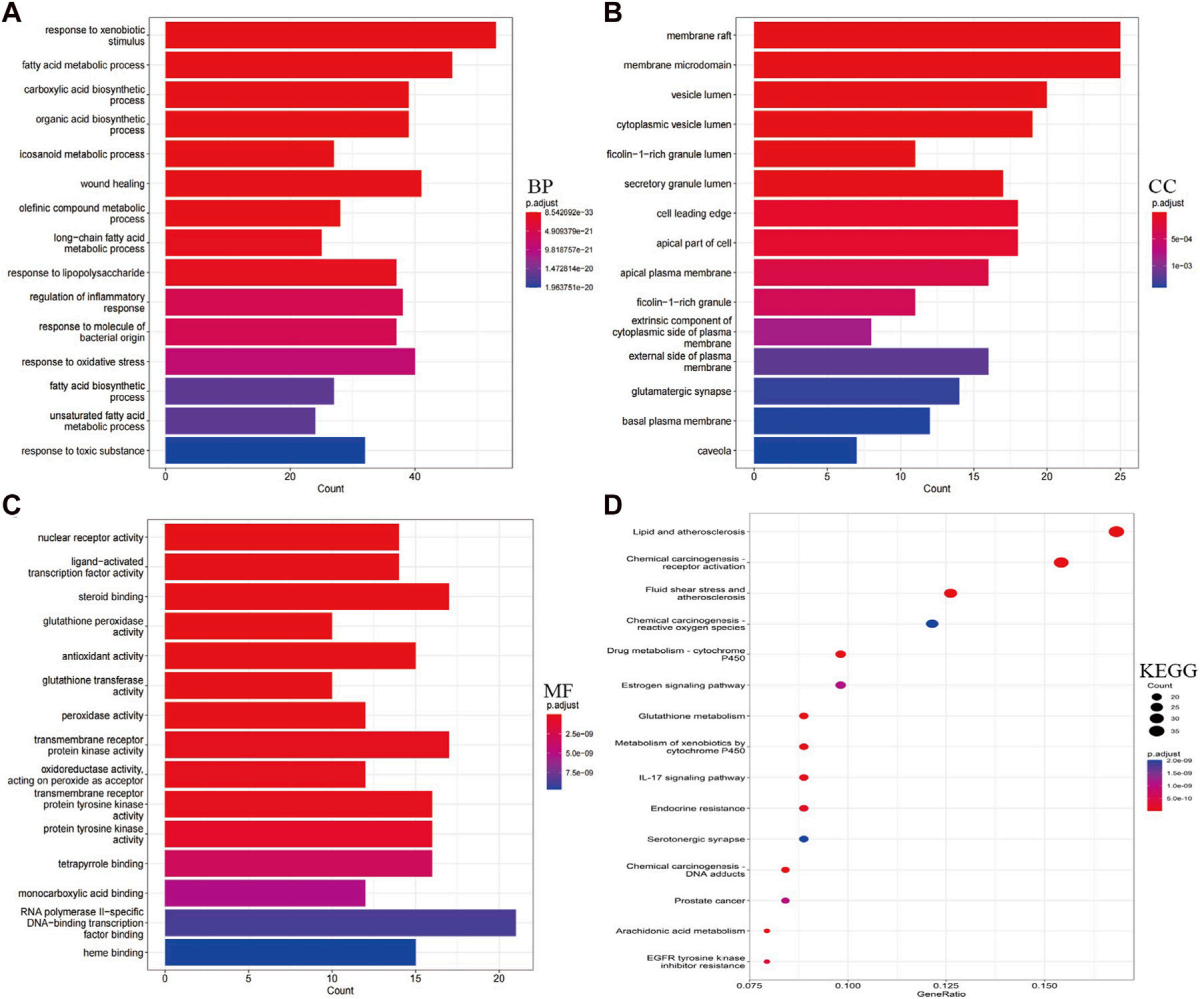
Display of enrichment analysis part results. **(A–C)** Based on Go enrichment analysis, functions in which garlic targets may be involved in AS, including biological process, cellular component and molecular function. **(D)** KEGG analysis results showed pivotal signaling pathways of garlic intervention in AS. The size of the bubble represents the number of genes, and the color of the bubble represents the size of the *p*-value. The darker the color, the smaller the *p*-value.

Using RStudio, GO and KEGG pathway enrichment analysis was performed on the 230 intersecting targets. The functional enrichment of genes was categorized into MF, CC and BP. A total of 2017 BPs, 78 CCs, and 200 MFs were obtained, with the top 15 significant themes for each module shown in Figure 3. GO-BP mainly included processes such as response to xenobiotic stimulus, fatty acid metabolic process, response to oxidative stress, and regulation of inflammatory response (Figure 3A). GO-CC comprised membrane raft, membrane microdomain, vesicle lumen, and cytoplasmic vesicle lumen, among others (Figure 3B). GO-MF included RNA polymerase II-specific DNA-binding transcription factor binding, steroid binding, transmembrane receptor protein kinase activity, and transmembrane receptor protein tyrosine kinase activity, among others (Figure 3C).

In addition, potential targets were mainly enriched in 172 KEGG pathways, including Lipid and atherosclerosis, Chemical carcinogenesis-receptor activation, Fluid shear stress and atherosclerosis, PI3K-Akt signaling pathway, MAPK signaling pathway, and Chemical carcinogenesis—reactive oxygen species (Figure 3D). Each signaling pathway is composed of different targets, with a particular focus on AS-related signaling pathways (Supplementary Figures S1, S2).

### 3.4 Active components of garlic—AS target interaction network

**FIGURE 4:**
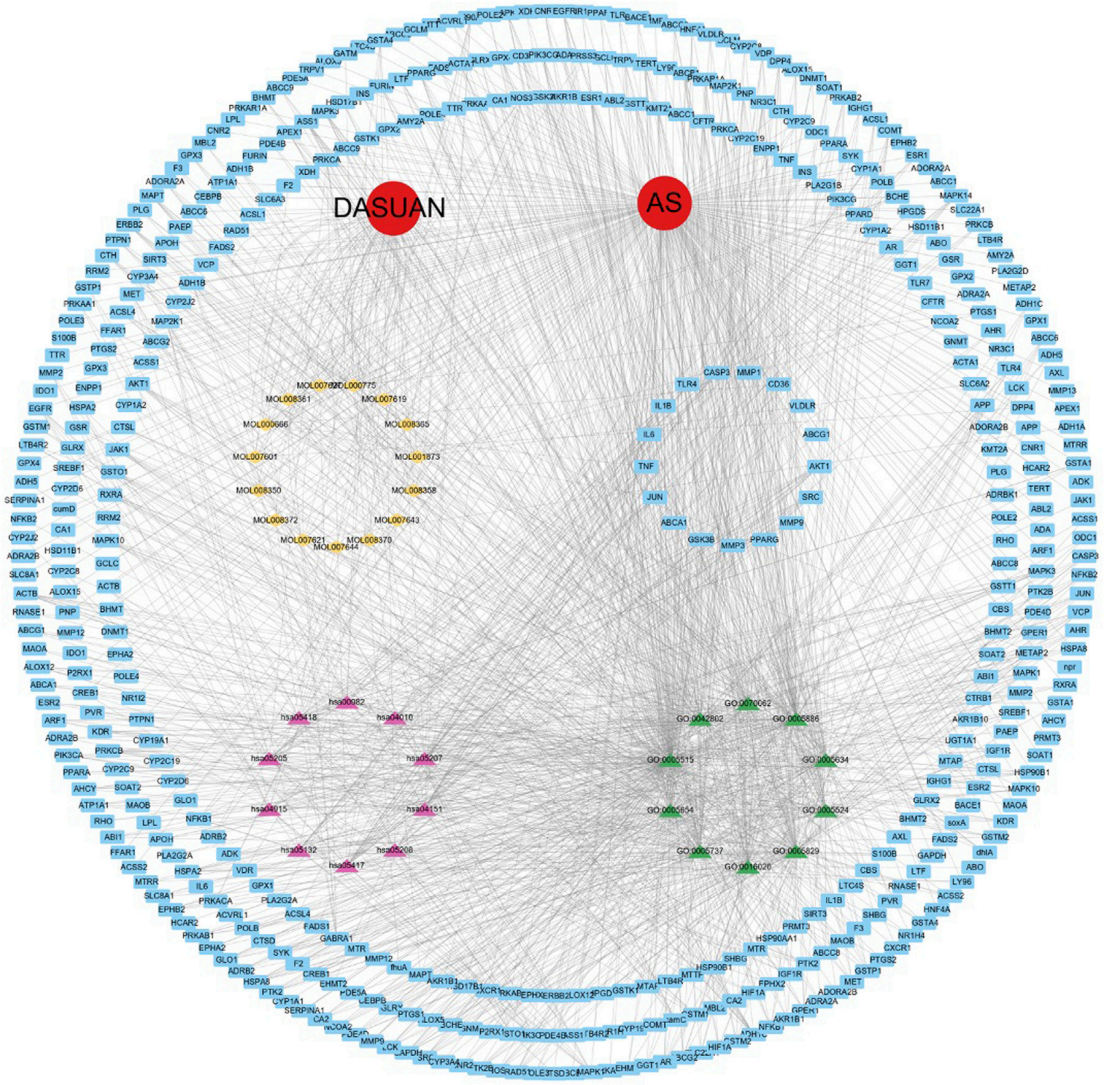
A network of garlic interventions for AS. The yellow marker is the main active ingredient of garlic. The blue markers are potential targets of action. The pink marker is the KEGG pathway. The green markers are GO analysis topics.

We imported active components, potential therapeutic targets, GO themes, and signaling pathways into Cytoscape software to generate the component-disease-target interaction network (Figure 4). In the network, the main targets are represented in blue, major active components of garlic in yellow, the top ten signaling pathways in pink, and the top ten GO themes in green. The red circles represent the key terms for both garlic and atherosclerosis.

### 3.5 Garlic may be involved in regulating the progression and stability of atherosclerotic plaques

**FIGURE 5:**
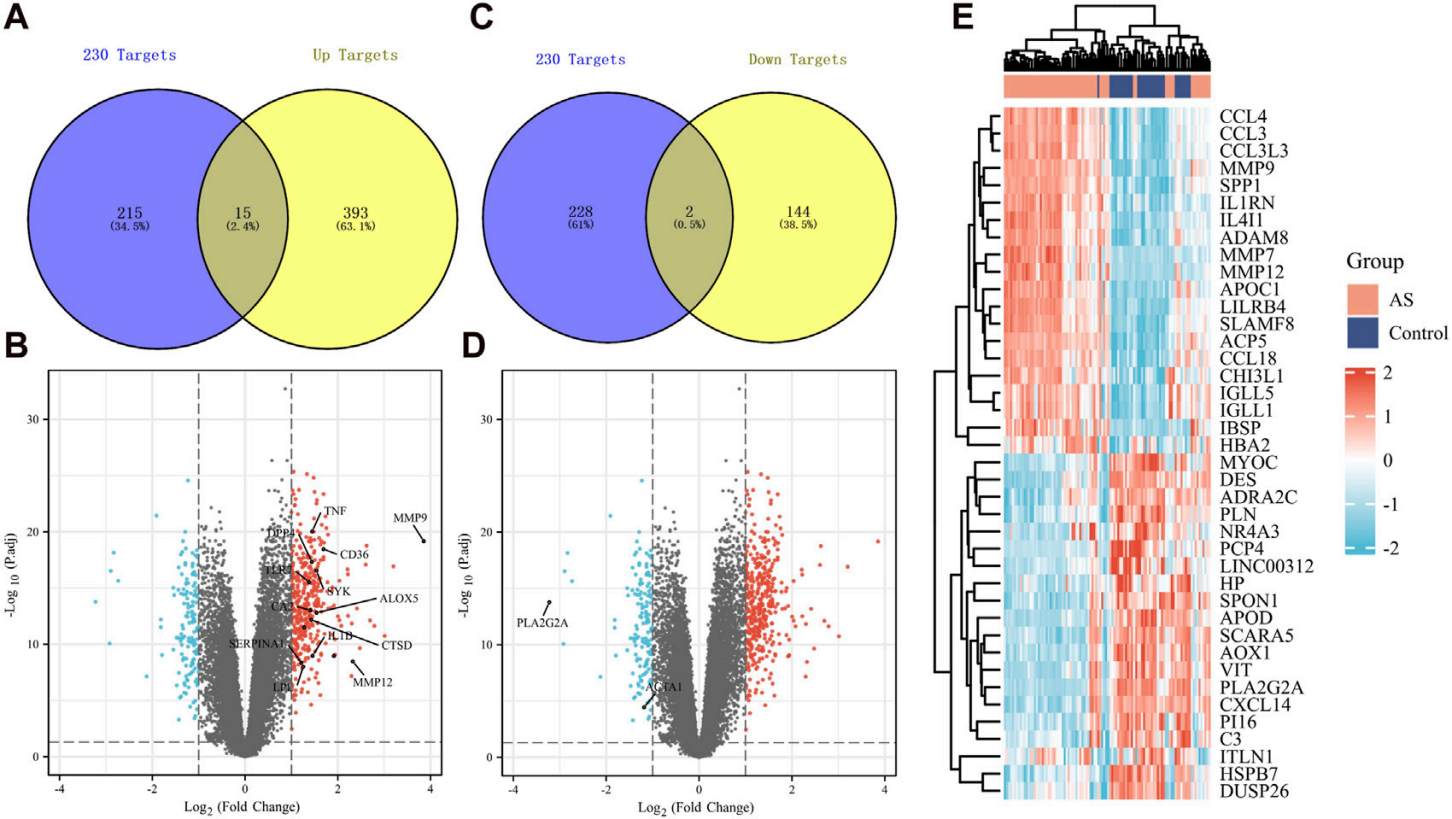
Expression of key genes in human tissues in AS. **(A,B)** 230 targets were intersected with upregulated differential genes, and 15 upregulated genes were obtained. **(C,D)** 230 targets were intersected with downregulated differential genes, and 2 downregulated genes were obtained. **(E)** Other potential key pathogenic genes in AS.

We obtained differentially expressed genes from human atherosclerotic plaques in the GSE100927 dataset. By intersecting with the previously mentioned 230 common genes, we identified 15 upregulated genes (Figures 5A, B) and 2 downregulated genes (Figures 5C, D). The upregulated genes include TNF, MMP9, DPP4, CD36, TLR7, SYK, CA2, ALOX5, CTSD, IL1B, SERPINA1, LPL, MMP12, RNASE1, and MMP1. The downregulated genes are ACTA1 and PLA2G2A. These genes may serve as potential targets for garlic in treating AS. Identified through literature searches and the FerrDb, TNF, MMP9, DPP4, CD36, ALOX5, IL1B, MMP12 and MMP1 have been found to be associated with ferroptosis. We reasonably hypothesize that the advancement of atherosclerosis is correlated with ferroptosis. Additionally, Figure 5E illustrates differentially expressed genes between vasculars of healthy individuals and those with atherosclerosis, which are also potential therapeutic targets.

We obtained differentially expressed genes that may cause intraplaque hemorrhage (IPH) from the GSE163154 dataset, representing crucial targets influencing plaque stability. By intersecting with the aforementioned 230 common genes, we identified 13 upregulated genes (Supplementary Figures S3A, B) and 3 downregulated genes (Supplementary Figures S3C, D). The upregulated genes include LY96, CA2, CTSD, MMP12, CD36, PPARG, GPX1, MMP9, LPL, RNASE1, ALOX5, IL1B, and ABCA1. The downregulated genes are HSPA2, MAOB, and PDE5A. These genes may be key targets for garlic in stabilizing atherosclerotic plaques and preventing IPH. MMP12, CD36, GPX1, MMP9, ALOX5, IL1B and PPARG have been found to be associated with ferroptosis. Once more, it demonstrates the significance of ferroptosis in plaque. Additionally, Supplementary Figure S3E represents differentially expressed genes between bleeding and stable plaques in patients, which are potential regulatory targets.

### 3.6 Garlic may improve atherosclerotic plaque by regulating ferroptosis

**FIGURE 6:**
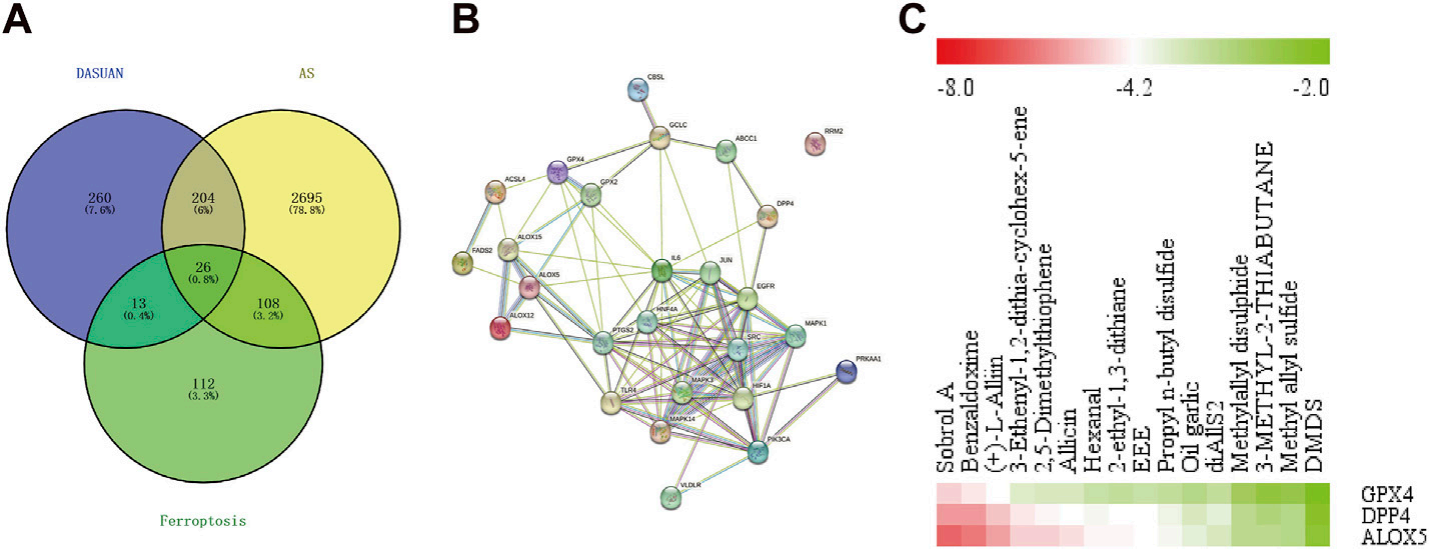
Garlic may improve atherosclerotic plaque by regulating ferroptosis. **(A)** The intersection of the targets of garlic, the targets of AS and the regulatory target of ferroptosis obtained 26 key genes. **(B)** The PPI network was obtained by importing 26 key genes into the String database. **(C)** Molecular docking is performed between garlic active components and ferroptosis-related genes GPX4, DPP4, and ALOX5, resulting in a heatmap of molecular binding energies.

We obtained related genes for ferroptosis from the FerrDb database and intersected them with targets of garlic and AS disease targets, resulting in 26 key genes (Figure 6A). Subsequently, we performed a protein-protein interaction (PPI) network analysis on these 26 target genes using the String database (Figure 6B). Through literature search, we identified GPX4, DPP4, and ALOX5 as key genes in ferroptosis, which have also been reported in AS damage studies. Molecular docking (Figure 6C) revealed that Sobrol A, Benzaldoxime, Allicin, and (+)-L-Alliin are the main active components in garlic. This suggests that garlic may improve AS progression by regulating ferroptosis.

### 3.7 Molecular docking verification

**FIGURE 7:**
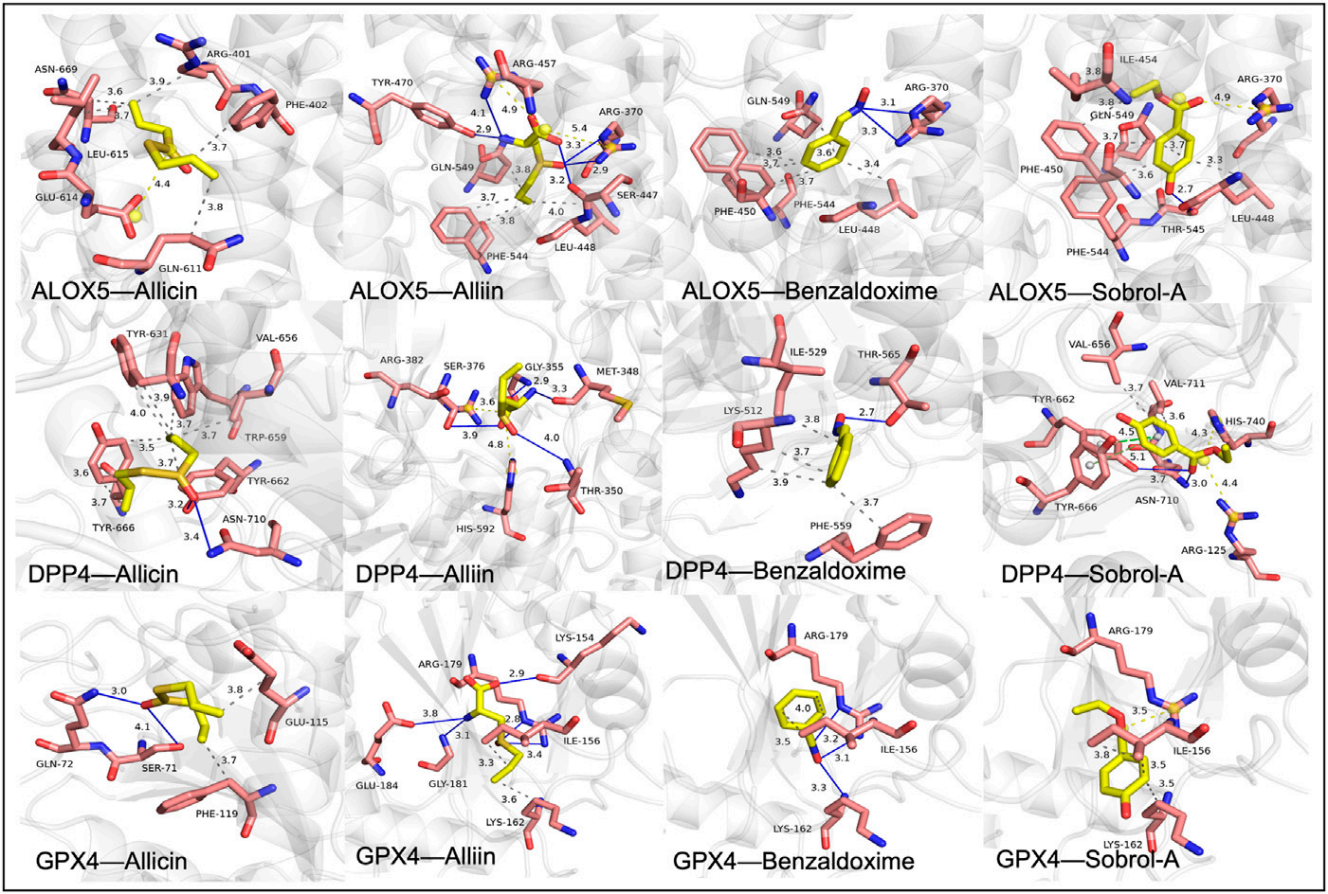
The interactions between protein receptors and ligands. Allicin binds to ALOX5, DPP4 and GPX4. (+)-L-Alliin binds to ALOX5, DPP4 and GPX4. Benzaldoxime binds to ALOX5, DPP4 and GPX4. Sobrol-A binds to ALOX5, DPP4 and GPX4.

**FIGURE 8:**
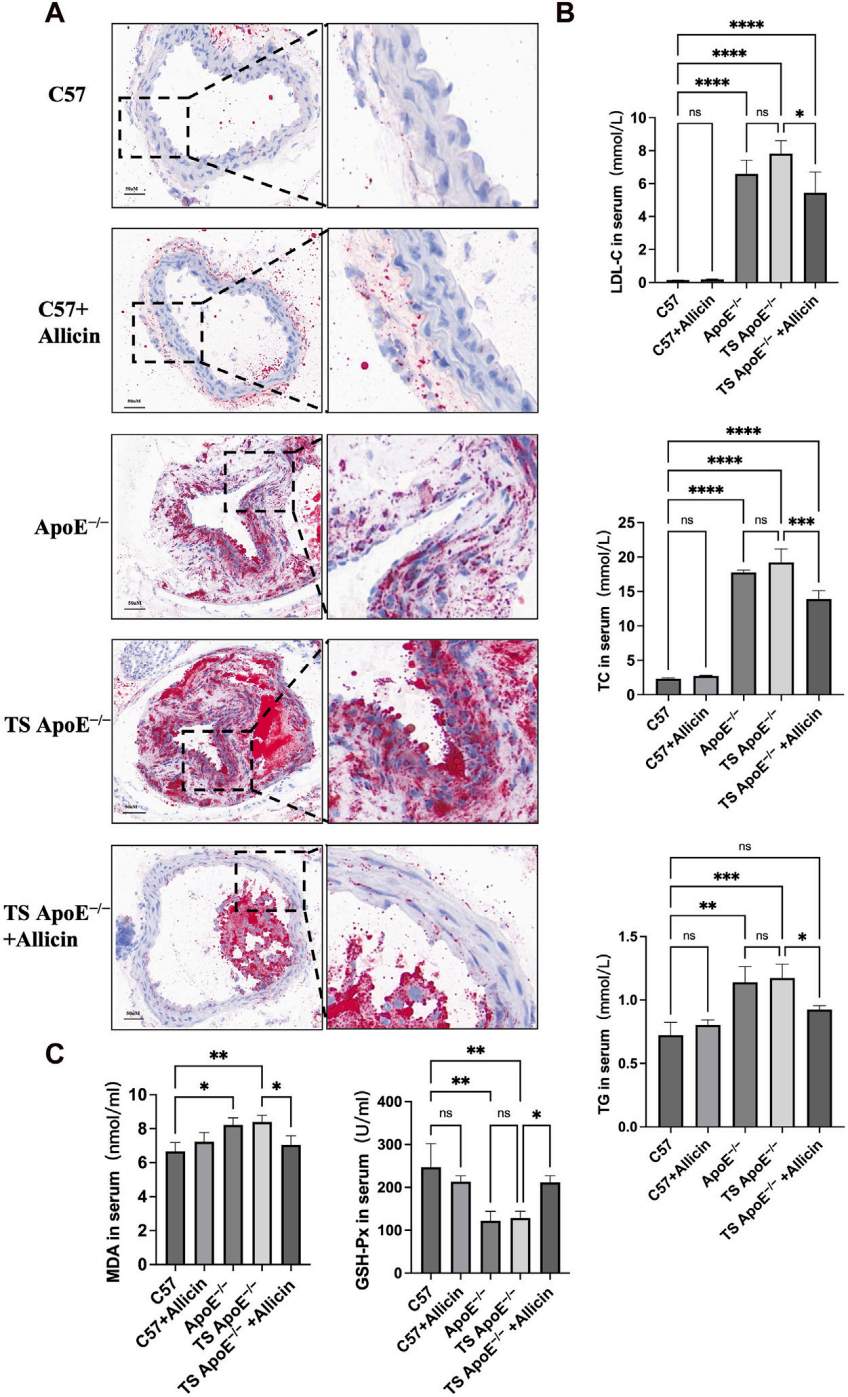
Animal experiment validation. **(A)** Oil red O staining examination was used to verify established carotid plaque (TS ApoE^−/−^) model and observed treatment effects. C57BL/6, C57BL/6+Allicin, ApoE^−/−^, TS ApoE^−/−^ and TS ApoE^−/−^ + Allicin groups. **(B)** LDL-C, TC and TG in serum were analyzed by biochemical detection. **(C)** The expressions of MDA and GSH-Px in serum were performed by detection kit. (*p < 0.05, **p < 0.01, ***p < 0.001, ****p < 0.0001, indicating statistically significant data between groups; ns indicates no statistical significance).

The stability of the binding conformation between receptors and ligands is inversely proportional to the molecular binding energy. We consider binding activities with energy levels below −4.2 kcal/mol to indicate meaningful interactions. Molecular docking was individually performed for the identified key genes and major active components, resulting in the determination of effective compounds in garlic. The molecular binding energy results are depicted in Figure 7 and detailed information is available in Table 2. The molecular docking outcomes are illustrated in Figure 8. Notably, Allicin binds to ALOX5, DPP4, and GPX4, while (+)-L-Alliin binds to ALOX5, DPP4, and GPX4. Benzaldoxime demonstrates binding with ALOX5, DPP4, and GPX4, and Sobrol-A exhibits binding with ALOX5, DPP4, and GPX4. This implies that garlic components such as Sobrol A, Benzaldoxime, Allicin, and (+)-L-Alliin exhibit strong binding activity with the aforementioned target proteins.

**TABLE 2:** Molecular docking results of core targets and active components.

	GPX4	DPP4	ALOX5
SobrolA	−4.8	−5.6	−6.4
Benzaldoxime	−4.5	−5.6	−6.1
(+)-L-Alliin	−4.2	−5	−5.5
3-Ethenyl-1,2-dithia-cyclohex-5-ene	−3.8	−4.5	−4.8
2,5-Dimethylthiophene	−3.7	−4.3	−4.8
Allicin	−3.7	−4.2	−4.6
Hexanal	−3.5	−4.1	−4.3
2-ethyl-1,3-dithiane	−3.4	−4.2	−4.3
EEE	−3.5	−4.2	−4.2
Propyl n-butyl disulfide	−3.4	−4	−4.1
Oil garlic	−3.2	−3.7	−3.9
diAllS2	−3.4	−3.9	−3.7
Methylallyl disulphide	−2.8	−3.3	−3.3
3-METHYL-2-THIABUTANE	−2.5	−3.1	−3.3
Methyl allyl sulfide	−2.6	−3.2	−3.2
DMDS	−1.9	−2.3	−2.4

### 3.8 Allicin treatment alleviated pathological changes and ferroptotic lipid peroxidation levels in TS Apoe^−/−^ mice

Histopathological examination utilizing Oil Red O staining facilitates the quantitative assessment of luminal area changes indicative of atherosclerotic plaque lesions. This further substantiates the protective effect of Allicin on carotid atherosclerosis. As depicted in Figure 8A, the carotid arteries in the C57 group exhibited a normal morphology with clear intimal boundaries, regular arrangement, and no observed vascular lesions. In contrast, the TS ApoE^−/−^ model group displayed significant intimal thickening, disrupted endothelial cell connections, and extensive lipid deposition. In terms of pharmacological treatment, the administration of Allicin ameliorated the pathological changes and mitigated lipid deposition in carotid artery plaque. Mouse lipid profiling was employed to evaluate the protective effect of Allicin against atherosclerotic disease. Figure 8B indicates that the Allicin group exhibited significantly lower concentrations of LDL-C, TC, and TG compared to the ApoE^−/−^ group. Additionally, MDA levels in the ApoE^−/−^ and TS ApoE^−/−^ groups were higher than in other groups, and GPX4 was lower compared to the remaining groups. Conversely, serum lipid peroxidation markers improved, and iron death levels were inhibited in the TS ApoE^−/−^ + Allicin group (Figure 8C). In summary, these results suggest that Allicin may regulate atherosclerotic lesions by inhibiting the iron death pathway, thereby preventing the occurrence of a stroke.

### 3.9 *In vitro* allicin intervention alters the survival status and ferroptosis levels in Raw264.7 cells induced by ox-LDL

**FIGURE 9:**
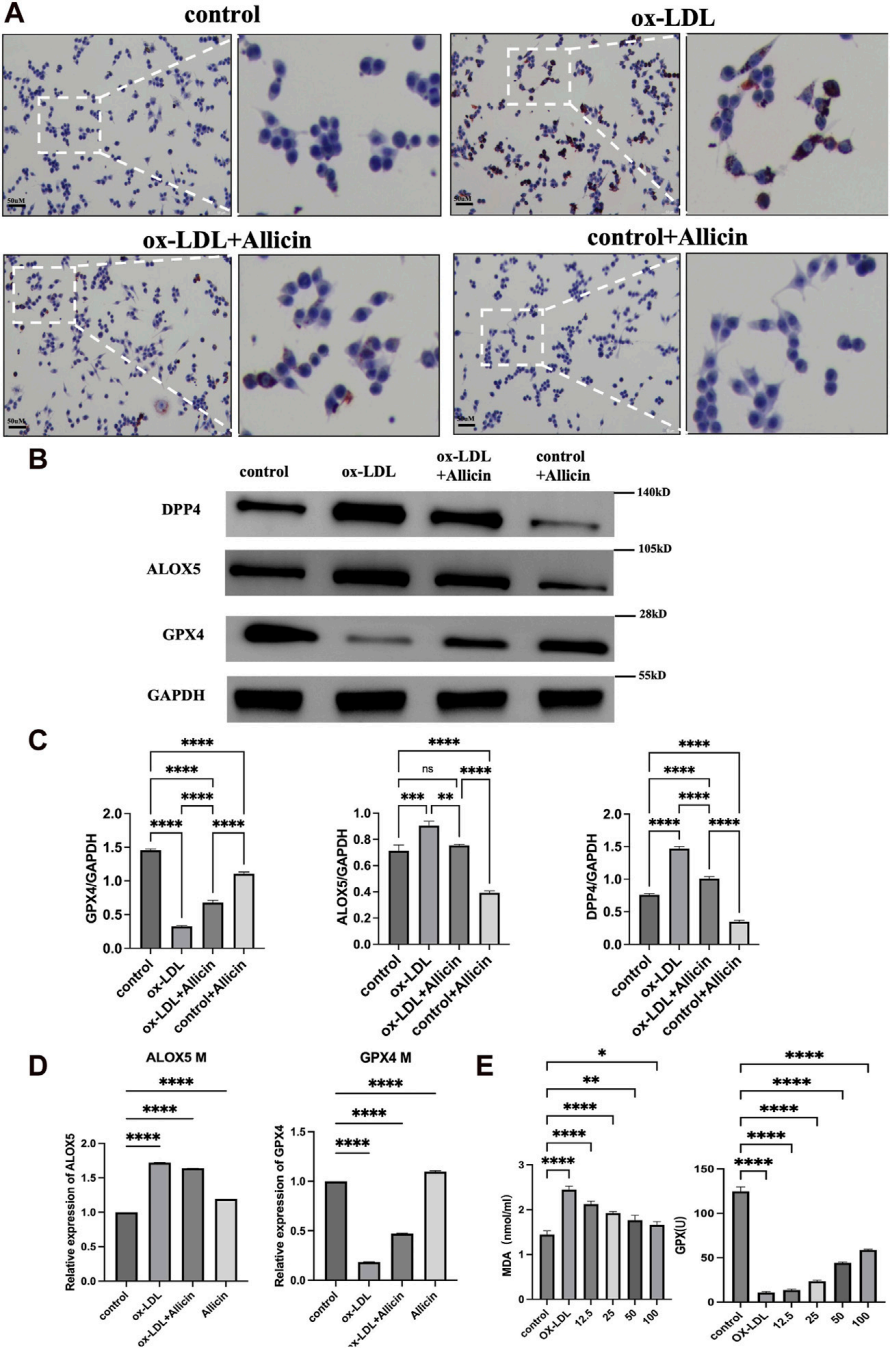
Cell experiment validation. **(A)** Oil red O staining was used to verify the treatment effect of Allicin in Raw264.7 induced by ox-LDL. Conteol, ox-LDL, ox-LDL + Allicin, Allicin groups. **(B)** The expressions of DPP4, ALOX5 and GPX4 protenin by western blots. **(C)** Represents the relative protein expression of GPX4, ALOX5 and DPP4. **(D)** Effect of Allicin on ALOX5 and GPX4 mRNA. **(E)** Effect of different concentration Allicin on MDA and GSH-Px content in Raw264.7 cells (*p < 0.05, **p < 0.01, ***p < 0.001, ****p < 0.0001, indicating statistically significant data between groups; ns indicates no statistical significance).

According to the preliminary experiment, we selected ox-LDL with a concentration of 80 mg/mL and Allicin with a concentration of 30 μg/mL for the subsequent experiment. Cell Oil Red O staining was employed to assess the impact of ox-LDL, Allicin, and ox-LDL + Allicin on Raw264.7 lesions (Figure 9A). The results indicated that cellular lipid damage in the ox-LDL group was more severe than in the control group. Administration of Allicin reduced oxidative lipid damage compared to the ox-LDL group. Protein expression analysis demonstrated that compared to the control group, the ox-LDL group exhibited higher protein expression of DPP4 and ALOX5, while the Allicin group showed reduced protein expression. The trend for GPX4 was exactly the opposite (Figures 9B, C). In conclusion, Allicin can improve ox-LDL-induced damage by inhibiting iron death levels. We used qRT-PCR to measure the effect of ox-LDL, Allicin, and ox-LDL + Allicin on the mRNA expression of ALOX5 and GPX4. The results showed that the mRNA expression levels of ALOX5 were higher in the ox-LDL group than in the control group (*p* < 0.05). After the addition of Allicin, the mRNA expression of ALOX5 was decreased compared with that in the ox-LDL group (*p <* 0.05). The mRNA expression trend for GPX4 was exactly the opposite (Figure 9D). Biochemical analysis of the cell culture supernatant revealed that the MDA content in the ox-LDL group was higher than in the other three groups (*p <* 0.05). Allicin administration reduced the MDA content. The GPX4 content in the Allicin group was higher than in the other three groups, indicating that Allicin reversed ox-LDL-induced macrophage lipid peroxidation damage (Figure 9E).

## 4 Discussion

Atherosclerosis is a chronic inflammatory disease driven by an imbalance in lipid metabolism and foam cell infiltration into the arterial wall. Its clinical symptoms are generally considered to be associated with the rupture of atherosclerotic plaque (Chiorescu et al., 2022). The rupture of atherosclerotic plaques is the most common cause of stenotic and occlusive diseases in the carotid and coronary arteries, manifesting as stroke and acute myocardial infarction (Libby, 2021).

In this study, 16 active components of garlic were screened, including Alliin, Allicin, Methylallyl sulfide, Methylallyl disulphide, DMDS, Benzaldoxime, 2-ethyl-1,3-dithiane, 3-Ethenyl-1, 2-dithia-cyclohex-5-ene, etc. The research indicates that garlic extract is associated with the progression of AS (Lu et al., 2023). Aged garlic extract inhibits progression of coronary artery calcification, lowers IL-6, glucose levels and blood pressure in patients at increased risk of cardiovascular events in a European cohort (Wlosinska et al., 2020). However, the specific mechanism by which garlic improves atherosclerosis remains to be elucidated.

In our work, we identified 503 potential targets of garlic, and by intersecting drug targets with atherosclerosis-related genes, we obtained 230 potential therapeutic targets. This suggests that nearly half of the garlic targets are associated with atherosclerosis. GO and KEGG pathway enrichment analysis of these targets revealed 200 biological functions and 172 signaling pathways directly involved in atherosclerosis, indicating potential mechanisms underlying therapeutic effect of garlic.

According to KEGG enrichment results, anti-AS effect of garlic may be related to pathways such as Lipid and Atherosclerosis, Chemical Carcinogenesis—Receptor Activation, Fluid Shear Stress and Atherosclerosis, PI3K-Akt Signaling Pathway, MAPK Signaling Pathway, and Chemical Carcinogenesis—Reactive Oxygen Species. In the Lipid and Atherosclerosis pathway, particularly elevated levels of LDL, are closely associated with atherosclerosis development (Libby, 2021). In atherosclerotic lesion, the uptake of LDL induces the transformation of macrophages into foam cells and low levels of HDL may associate with reduced cholesterol efflux from foam cells, aggravating atherosclerosis (Tall et al., 2022). A meta-analysis of the impact of garlic on serum TC in a mouse model of hyperlipidemia showed that garlic effectively reduced TC, TG, and LDL-C concentrations while increasing macrophage activity and T cell, B cell production, showing immunomodulatory effects (Li M. et al., 2022; Osadnik et al., 2022; Fu et al., 2023). The expression of scavenger receptors, SR-A and CD36, directly influences lipid deposition in macrophages, affecting foam cell formation (Shu et al., 2022). Garlic compounds significantly inhibit the activation of JNK and p38MAPK, thereby downregulating SR-A and increasing CD36 expression, impacting foam cell formation (Li M. et al., 2022). Garlic induces upregulation of ABCA1, reducing lipid accumulation in foam cells derived from THP-1 macrophages by activating the PPARγ/LXRα signaling pathway (Lin et al., 2017). Reactive oxygen species (ROS) play a crucial role in AS progression. Garlic has been shown to improve anti-oxidant effect by increasing total antioxidant capacity (T-AOC) and decreasing MDA levels (Moosavian et al., 2020). In advanced plaque, foam cells further progress to ferroptosis, forming characteristic necrotic core and exacerbating vulnerable plaques, suggesting that ferroptosis is the potential value target in atherosclerosis (Bai et al., 2020).

Through bioinformatic analysis, we intersected the DEGs obtained from clinical RNA-seq in atherosclerosis with the identified 230 target genes, resulting in the identification of 15 upregulated genes and 2 downregulated genes. Notably, 8 out of the 15 DEGs were found to be associated with ferroptosis. Among these, TNF, CD36, DPP4, and ALOX5 can promote ferroptosis by inducing the production of reactive oxygen species (ROS) and lipid peroxidation (Tang et al., 2021; Wu et al., 2022). MMP9, MMP1, and MMP12 can indirectly affect ferroptosis by influencing the cellular microenvironment and pro-inflammatory responses (Liu et al., 2023). Additionally, IL-1β may promote ferroptosis by activating pro-inflammatory signaling pathways and increasing ROS levels (Chen et al., 2023). Consequently, we reasonably infer that ferroptosis plays a pivotal role in the intervention of garlic for atherosclerosis.

To validate the potential therapeutic efficacy of garlic components, we utilized the AutoDock Vina software, focusing on the core ferroptosis targets, namely, DPP4, ALOX5, and GPX4. The molecular docking results revealed that the active components, Sobrol A, (+)-L-Alliin, Benzaldoxime, and Allicin, exhibited strong binding affinities with GPX4, DPP4, and ALOX5, consistently achieving docking strengths below −4.2 kcal/mol. Furthermore, Allicin demonstrated stable binding with DPP4 and ALOX5. These findings suggest that Sobrol A, (+)-L-Alliin, Benzaldoxime, and Allicin present in garlic represent potential active compounds for the treatment of atherosclerosis.

In the experimental validation using a ApoE^−/−^ model with carotid artery ligation and Raw264.7 cell experiments, the administration of garlic through gastric lavage resulted in a significant improvement in carotid plaque. Serum MDA and GSH-Px indicated a reduction compared to the plaque group, suggesting that garlic may inhibit the progression of atherosclerosis by suppressing ferroptosis and reducing lipid peroxidation.

In conclusion, we identified the targets of ferroptosis in atherosclerosis associated with garlic, including GPX4, DPP4 and ALOX5, providing new mechanistic insights that may be clinically relevant for combination therapies of garlic. The active components of garlic, specifically Sobrol A, (+)-L-Alliin, Benzaldoxime, and Allicin, demonstrate the potential to emerge as promising agents for future treatment targeting atherosclerotic diseases.

## Data Availability

The original contributions presented in the study are included in the article/Supplementary Material, further inquiries can be directed to the corresponding authors.

## Supplementary material

SUPPLEMENTARY FIGURE S1 The lipid and atherosclerosis signaling pathway. Arrows indicate activation effects; the red targets are the pathways we focused on in this study.

SUPPLEMENTARY FIGURE S2 The fluid shear stress and atherosclerosis.

SUPPLEMENTARY FIGURE S3 Garlic stabilizes AS by reducing IPH. **(A,B)** 230 targets were intersected with up-regulated differential genes, and 13 up-regulated genes were obtained. **(C,D)** 230 targets were intersected with down-regulated differential genes, and 3 down-regulated genes were obtained. **(E)** Other potential key pathogenic genes in AS.
