# Supplementary figures and images for: Garlic ameliorates atherosclerosis by regulating ferroptosis pathway: an integrated strategy of network pharmacology, bioinformatic and experimental verification
Source: Front Pharmacol. 2024 Jul 23;15:1388540. doi: 10.3389/fphar.2024.1388540 (PMC11300214; doi:10.3389/fphar.2024.1388540)

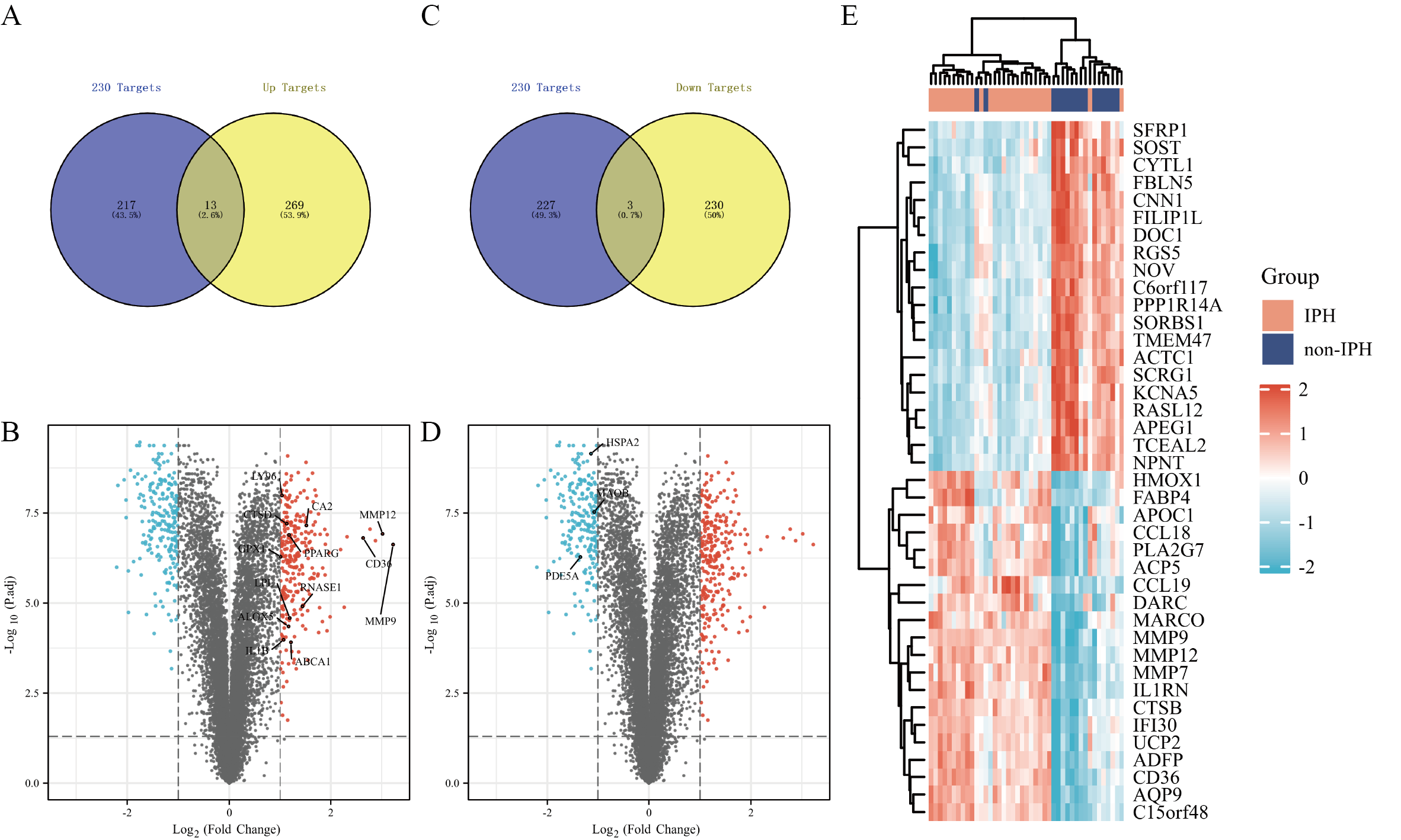

Supplement: Supplementary file 1 [file Image3.JPEG]

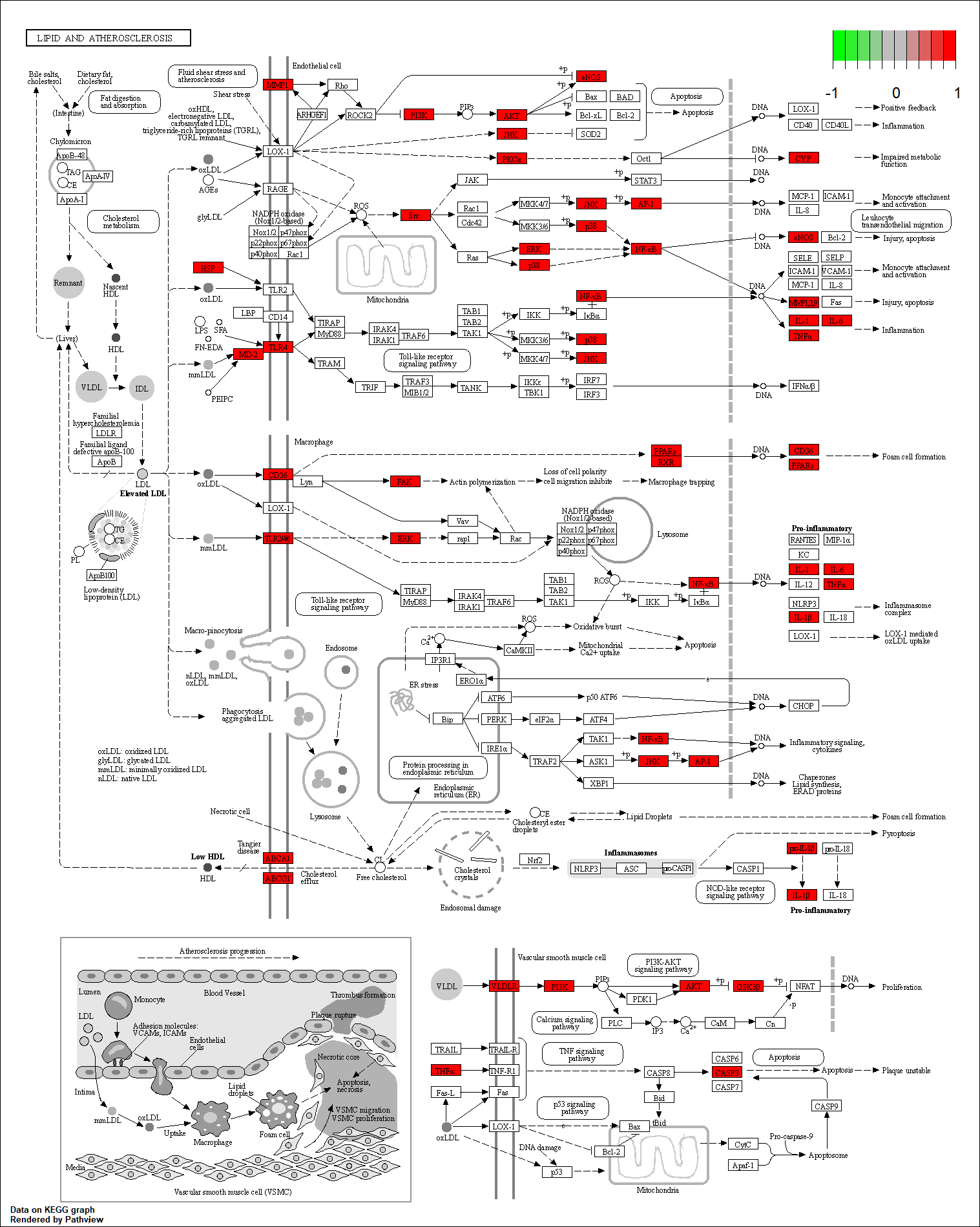

Supplement: Supplementary file 2 [file Image1.JPEG]

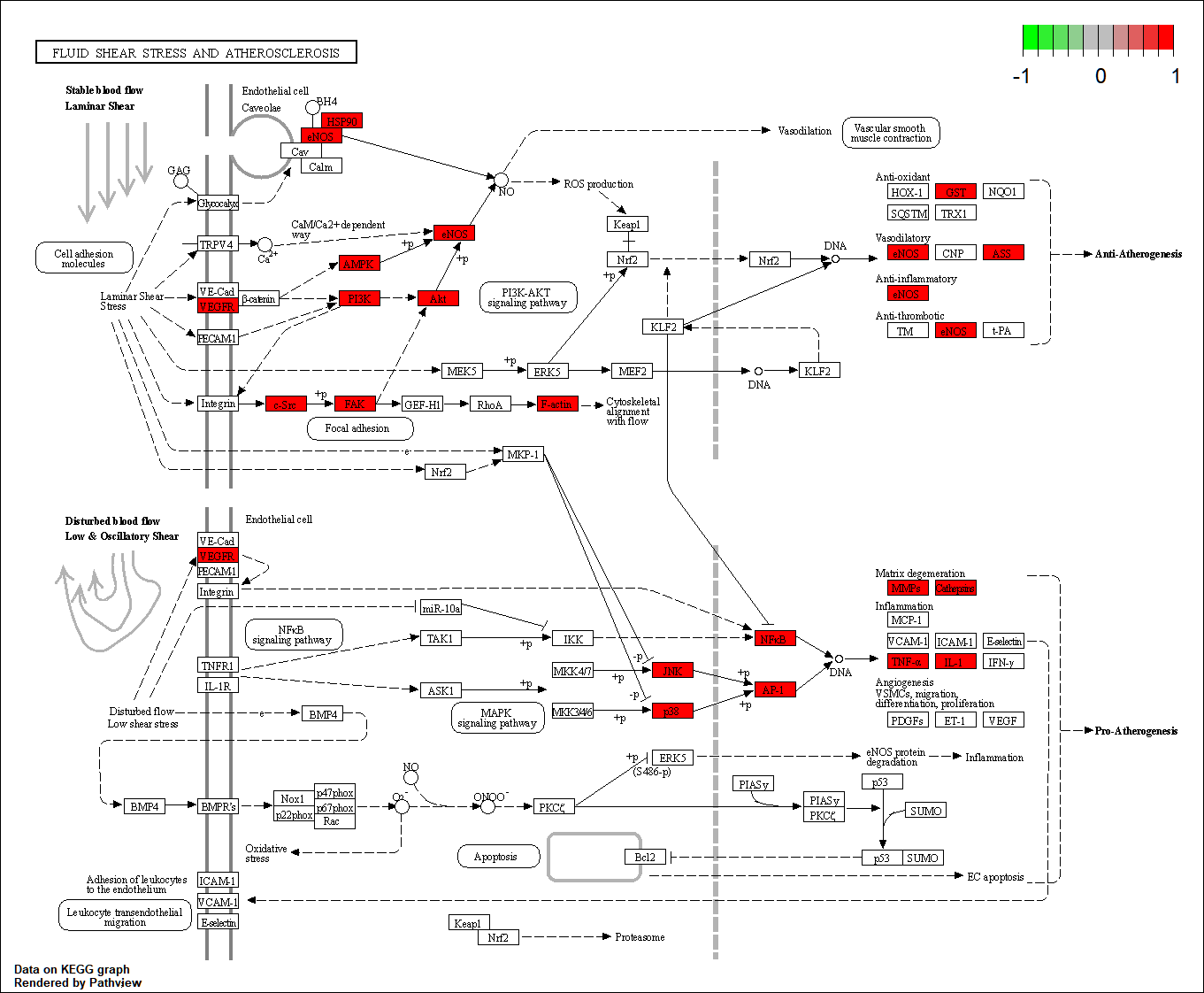

Supplement: Supplementary file 3 [file Image2.JPEG]
